# Light them up: photoresponsive imine-containing systems

**DOI:** 10.1039/d5sc05210f

**Published:** 2025-09-01

**Authors:** Jiarong Wu, Jake L. Greenfield

**Affiliations:** a Institut für Organische Chemie, Universitat Würzburg 97074 Würzburg Germany Jake.Greenfield@uniwuerzburg.de; b Center for Nanosystems Chemistry (CNC), Universitat Würzburg 97074 Würzburg Germany

## Abstract

The reversible covalent bond formation that underpins dynamic covalent chemistry (DCC) enables the construction of stimuli-responsive systems and the efficient assembly of complex architectures. While most DCC studies have focused on systems at thermodynamic equilibrium, there is growing interest in systems that operate away from equilibrium—either by shifting to a new free-energy landscape in response to a stimulus, or by accessing an out-of-equilibrium state following an energy input. Imine-based systems are especially attractive due to the accessibility of their building blocks and their dynamic behavior in both condensation and transimination reactions. These equilibria can be perturbed by chemical stimuli or light. While many modular systems combining imines with separate photoswitches have been studied in the context of light-responsive DCC, only recently have imine-based photoswitches—where light responsiveness is built directly into the dynamic covalent bond—emerged as a distinct strategy. In this perspective, we compare representative examples of both approaches, outline their respective strengths, and discuss key challenges and opportunities for advancing light-driven, out-of-equilibrium imine systems.

## Abstract 2

Imines, a type of dynamic covalent bond, are widely used in responsive materials. Recently, interest has grown in photoresponsive imine systems. Here, we share our perspective on two approaches to render imine systems photoresponsive.

## Graphical abstract

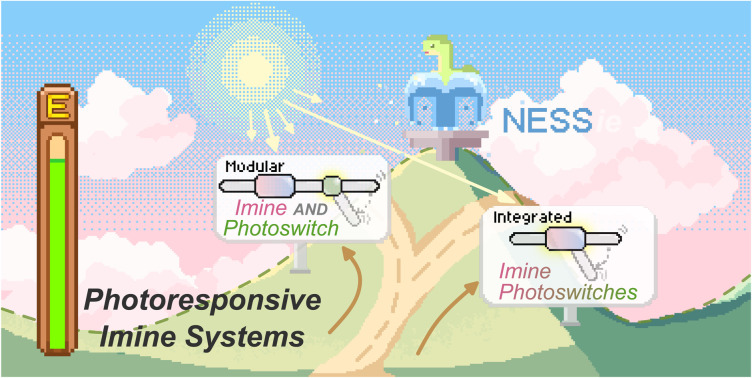

## Introduction

**Fig. 1:**
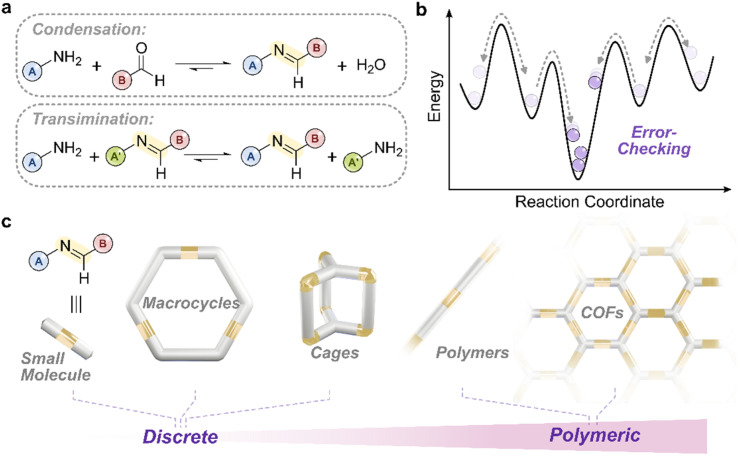
(a) Reversible imine formation and exchange *via* condensation and transimination reactions. Labels A, A′, and B indicate the amine and aldehyde components. Condensation proceeds *via* a tetrahedral hemiaminal intermediate, while transimination proceeds *via* a tetrahedral aminal intermediate. (b) Schematic free-energy landscape illustrating the error-checking mechanism in dynamic covalent systems. Reversible bond formation allows intermediates to be biased toward the thermodynamic product. (c) Representative imine-based architectures formed under thermodynamic control, including macrocycles, cages, and extended polymeric networks.

Dynamic-covalent chemistry (DCC) typically involves the reversible formation and cleavage of covalent bonds under thermodynamic control,^1–3^ in contrast to the kinetic control that governs conventional, irreversible covalent bond formation and cleavage.^4^ The use of dynamic-covalent (DC) linkages enables the construction of complex and stimuli-responsive architectures with relative synthetic ease,^2^ due to an inherent error-checking mechanism that drives systems toward their thermodynamic minimum state (Fig. 1).^2,4,5^ As a result, DCC has been applied to a wide range of areas of chemistry from the fabrication of molecular and macroscopic self-assembled materials to developing sensors and artificial molecular machines.^2,6,7^ Traditionally, DC systems have been studied at equilibrium, where the product distribution reflects the underlying free energy landscape. More recently, however, there has been growing interest in exploring DC systems that can be perturbed away from equilibrium^1,8^—either by applying a stimulus that shifts the system to a new free-energy landscape, or accessing out-of-equilibrium states through an energy input—thereby enabling new structures, behaviors and functions to emerge.^1,5,9–13^

Among the most studied DC motifs, imines stand out for their versatility and accessibility.^4,14–16^ They have been widely employed in structures ranging from small molecules^16–18^ to discrete assemblies such as macrocycles^19–24^ and cages,^14,25–31^ as well as extended polymeric^19,32–35^ and framework materials (Fig. 1c).^36–38^ Imines are dynamic in both their formation (*via* the condensation of amines with aldehydes) and their exchange (transimination) reactions.^39,40^ This dual dynamicity allows imine-containing systems to respond to various stimuli, including chemical additives and light, making them excellent candidates for designing systems that can be perturbed, and operated, out of equilibrium (OOE).^1^

In this perspective, we examine how light can serve as a stimulus to drive imine-based systems OOE. We compare two design approaches: (1) modular systems, in which imine bonds and light-responsive motifs are relatively separate units, and (2) integrated systems, where the imine bond itself acts as both the dynamic-covalent and photochromic unit. Through representative examples, we highlight the key differences between these approaches, explore their respective advantages and limitations, and outline open questions and challenges for the development of light-induced, OOE imine-based systems.

## From thermodynamic equilibria to light-induced non-equilibrium states

### Thermodynamic control in dynamic covalent imine chemistry

Imines—specifically aldimines (R^1^HC=NR^2^), formed *via* the reversible condensation of primary amines with aldehydes through an sp^3^ hemiaminal intermediate (Fig. 1a)—are among the most accessible and widely used DC linkages.^40^ Their formation is governed by thermodynamic control: the system evolves toward the lowest free-energy state, with the reversibility of the imine bond enabling the error-correction of on-pathway intermediates and off-pathway species trapped in local minima (Fig. 1b).^40^ This reversibility has made imines highly effective for assembling complex and stimuli-responsive architectures from judiciously designed building blocks, often in high yields and with minimal synthetic effort.^41–43^ Such structures include organic and metal–organic cages—^44,45^ useful for molecular sequestration and sensing—as well as polymeric materials of varying dimensionality for applications in (opto)electronics^32,46–48^ and materials science.^41^

In addition to reversible condensation and hydrolysis, imines also exhibit dynamicity in transimination reactions:^39^ an exchange reaction in which an imine reacts with a competing amine to form a new imine, proceeding *via* a sp^3^ aminal intermediate, and releasing the original amine component of the imine.^39,49^ The nucleophilicity of the incoming amine influences the rate of transimination, while the final product distribution reflects the thermodynamic stability of the system. In many cases, more nucleophilic amines also give rise to the most thermodynamically stable imines, aligning kinetic and thermodynamic preferences. However, this preference can be modulated. For example, addition of an equivalent of acid to protonate the more basic (and typically the more nucleophilic) amine decreases its nucleophilicity, thus altering the hierarchy of amine preference.^50,51^ This tunability under thermodynamic control allows the formation of diverse stimuli-responsive structures. Accordingly, the study of imine-based dynamic-covalent systems has remained a focus for many research groups, including Lehn,^24,52,53^ Barner-Kowollik,^48,54^ Di Stefano,^50,51^ Hecht,^55^ You^56,57^—and has been the subject of several comprehensive reviews.^2,7,40,58^

**Fig. 2:**
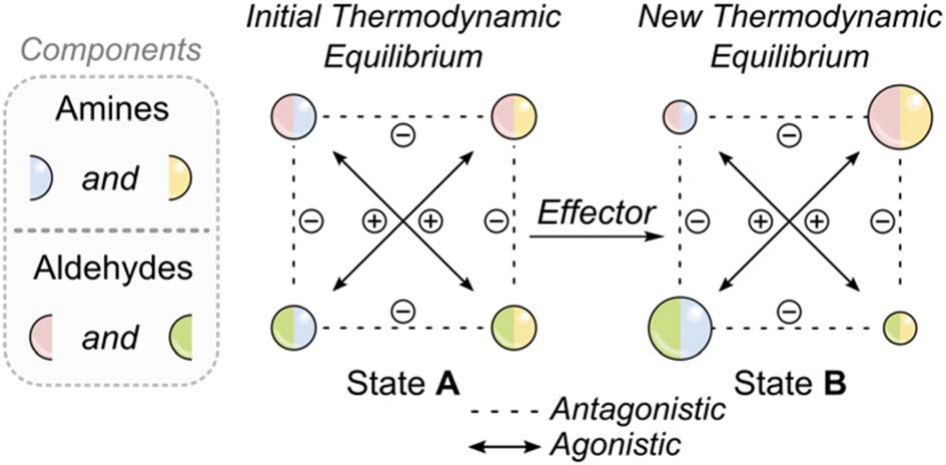
Schematic of a dynamic covalent reaction network, as pioneered by Lehn and co-workers. The system comprises four interconverting imines (represented as bicolored spheres), formed from two amines (blue and yellow) and two aldehydes (green and red). State A illustrates the initial distribution, which is nearly statistical due to similar component reactivities, reflecting the system's thermodynamic equilibrium. Upon addition of an effector that selectively stabilizes the red-yellow imine, the system shifts to minimize its free energy by forming more of this component. This shift consumes imines containing either red or yellow fragments (antagonistic interactions), releasing blue and green components, which combine to form more of the blue-green imine (agonistic interaction). Overall, the effector reshapes the free-energy landscape, and the system responds by adopting a new thermodynamic distribution.

The reversible and adaptive nature of imine systems has also enabled the construction of dynamic covalent networks composed of interacting and interdependent components.^24,59–62^ This area—extensively pioneered by Lehn and co-workers—^63–65^ falls under the broader umbrella of systems chemistry: the study of emergent properties and behaviors that arise at the “systems level” from the interaction and interdependence of system components.^66^ For example, combining two different aldehyde components with two different amines generates a mixture of four distinct imines—a dynamic covalent library (DCL, Fig. 2).^63,67,68^ The statistical distribution of imines in this library reflects the underlying thermodynamic landscape of the “system”: since the imines compete for shared building blocks, the thermodynamic preferences of the system as a whole dictate the product distribution. This interdependence between imines gives rise to agonistic and antagonistic behavior,^69^ where changes in the population of one imine in the system affect the others.

**Fig. 3:**
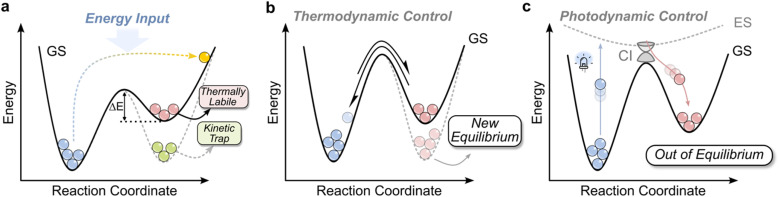
(a) Overview of different OOE states. If the barrier for returning to equilibrium is >>*k*_B_*T*, the system becomes kinetically trapped; if ∼*k*_B_*T*, it is thermally labile. Such a thermally labile metastable state is an example of a dissipative state, requiring continuous energy input to be maintained. (b) Under thermodynamic control, the addition of an effector modifies the system's energy landscape, shifting the equilibrium distribution to favor a new minimum. (c) Under photodynamic control, photoexcitation allows the system to escape equilibrium. In a two-state photoswitch, vertical excitation from the ground state (GS) to the excited state (ES), followed by relaxation through a conical intersection (CI), funnels the molecule to either the thermodynamically stable (blue) or metastable (red) isomer. Differences in extinction coefficients and quantum yields at the irradiation wavelength result in an OOE distribution of isomers. Under continuous irradiation, this establishes a steady-state distribution, a PSS.

An interesting question arises: how can such a DCL be perturbed from this equilibrium state, and how do these perturbations propagate across the network? In this context, Lehn introduced the concept of constitutionally dynamic systems,^70^ which respond to effectors—external physical or chemical stimuli that selectively stabilize or destabilize certain components of the network (Fig. 2).^24,62,65,71^ These effectors modify the relative depths of energy minima on the system's free-energy surface (*vide infra*Fig. 3b), displacing the system from its initial equilibrium and prompting a shift toward a new thermodynamic minimum. This concept of dynamic adaptation has been instrumental in designing systems that respond to physical and chemical changes.^72,73^ If the effector can be removed or consumed, the system can return to its original state, enabling reversible and cyclic control over the distribution of network components.^74^

### Escaping thermodynamic equilibrium: the role of light

As noted above, imine-based systems are typically studied under equilibrating conditions, where product distributions reflect the underlying thermodynamic landscape. However, there is growing interest in driving these systems away from equilibrium—at least temporarily—to access new structures and functions inaccessible under equilibrium control.^75^ These OOE states can be broadly classified by their longevity and how they are maintained (Fig. 3a):^1^ from long lived kinetically trapped states (where the activation barrier to return to equilibrium is >>*k*_B_*T*), to thermally labile states (Δ*E* > *k*_B_*T*). The latter is an example of a dissipative state and requires a continuous energy input to persist. It can be visualized as blowing a ball uphill: once the energy input stops, the system rolls back to equilibrium, dissipating potential energy in the process.

In dissipative systems, a non-equilibrium steady state (NESS) can be established by continuously supplying energy to the process of interest to keep it out of equilibrium.^1,76^ A macroscopic analogy of a NESS is a water fountain: the shape of the water stream appears stable but only exists due to the continuous action of a pump. Once the energy input stops, the shape collapses and equilibrium is restored. NESSs are thus defined by sustained flux and energy dissipation, achieved by a continuous energy input. Systems at a NESS are particularly interesting for their potential to couple the energy dissipation process to one that produces useful work, continuously.^76^

A molecular-level example of a NESS is a photoswitch—a molecule that can convert between two or more states in response to light—that is operating under constant light irradiation, and where the metastable state is thermally labile (Fig. 3a). Because the different states of the switch can differ in extinction coefficient and quantum yield, the steady-state populations under irradiation deviate from the Boltzmann distribution (Fig. 3b and c). This gives rise to a light-driven, non-equilibrium distribution known as a photostationary state (PSS), which is a NESS (Fig. 3c). If the irradiation is paused, these thermally labile switches will revert back to their equilibrium distribution, thus constant irradiation is required to maintain the NESS. However, if instead the light-induced state is kinetically trapped, the system can remain at the photostationary distribution for a period of time and this situation does not correspond to a NESS, as there is no flux in the system. In the following discussion, the metastable state, which includes both thermally labile states and kinetically trapped states, will be used generally to refer to the light-induced state of a photoswitch, unless specified.

Compared to thermodynamic control, photodynamic control enables light-driven systems to escape detailed balance (Fig. 3b and c). While essentially every reversible photoswitch can reach a PSS under irradiation, it arguably only becomes functionally interesting when it can be coupled to a thermally driven reversible process, such as imine exchange, whose equilibrium is asymmetrically influenced by the switching event.^76^ In this way, the light input can be coupled to drive a chemical change—a central goal in the development of light-powered molecular machines.

Driving a system OOE—whether achieved chemically or photochemically—requires breaking detailed balance, the equilibrium condition ensuring that no net flux occurs between the states.^10^ In photochemical systems, detailed balance is broken when light interacts differently with each state—typically because the isomers have distinct extinction coefficients or quantum yields at the irradiation wavelength. Having escaped detailed balance using light, the directionality exhibited by the system then typically emerges from kinetic asymmetry in the thermal exchange process—a directional bias in the pathways across the energy landscape.^77^ This concept, theoretically pioneered by Astumian^77,78^ and experimentally studied by Leigh,^10,79^ underpins the operation of chemically driven and light-fueled systems. For light-driven systems, the key challenge is not simply perturbing a reversible photochemical reaction with light,^76^ but coupling this light-induced change to an equilibrium process—such as imine exchange—in a way that imposes a directional flux (kinetic asymmetry). As Aprahamian and Goldup have pointed out, this coupling is critical for turning photochemical input into useful work.^76^

In summary, dynamic imine systems provide a reversible and responsive platform that can be perturbed from thermodynamic equilibrium by external stimuli. Light, as a clean, traceless, and tunable energy source, enables access to light-dependent OOE states. While NESSs are inherent to thermally labile photoswitches, the real interest and opportunity arguably lies in coupling these light-induced perturbations to dynamic covalent exchange—driving a thermal reaction OOE. This coupling is challenging and a primary focus of current efforts in the field.

### Design strategies for photoresponsive dynamic imine systems

**Fig. 4:**
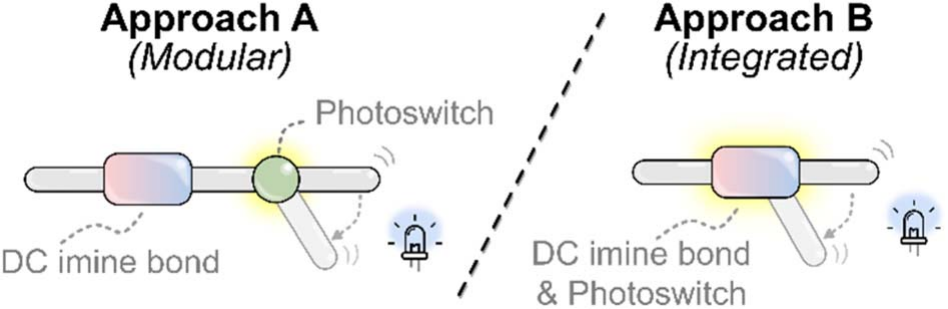
Design strategies for light-responsive imine systems. Approach A (modular): the photoswitch and imine are incorporated as distinct but functionally coupled units, allowing each to be tuned more independently. Approach B (integrated): the imine bond itself acts as the light-responsive unit, combining the dynamic-covalent linkage and photochromic behavior into a single motif.

To design dynamic covalent systems in which light can drive imine-containing systems out of equilibrium, two general strategies can be employed (Fig. 4): (A) a modular strategy, in which the imine and photoswitch are distinct but functionally connected; and (B) an integrated strategy – in which the imine bond itself serves as the photoresponsive unit. The following sections explores representative examples of both approaches, drawing comparisons between their mechanisms, behaviors, and design implications. While light can also indirectly influence imine exchange by modulating pH *via* photoacids or photobases, this aspect lies beyond the scope of this discussion; for relevant reviews, we refer the reader to the following ref. 80–82.

### Modular systems: distinct photoswitch and imine motifs

In the modular approach, the photoswitch and the dynamic-covalent imine bond are distinct units, though they may still interact sterically or electronically. The photoswitch functions as a light-responsive effector that modulates imine assembly or exchange, typically by altering the local steric environment or electronic properties at the imine bond. This strategy offers design flexibility: the photochromic and dynamic-covalent components can be tuned relatively independently—for example, by optimizing the switching completeness and efficiency or the absorption wavelength of the photoswitch, or by selecting different partners for imine exchange.

When the photoswitch and imine are electronically conjugated (*i.e.*, part of the same π-system), photoisomerization can directly modulate the electronics of the imine bond. In more electronically decoupled systems, structural changes induced by switching—such as changes in geometric requirements or variations in sterics—can also perturb imine reactivity. The following sections explore representative examples illustrating these different modes of influencing the imine bond.

### Light-mediated electronic control of imine reactivity

**Fig. 5:**
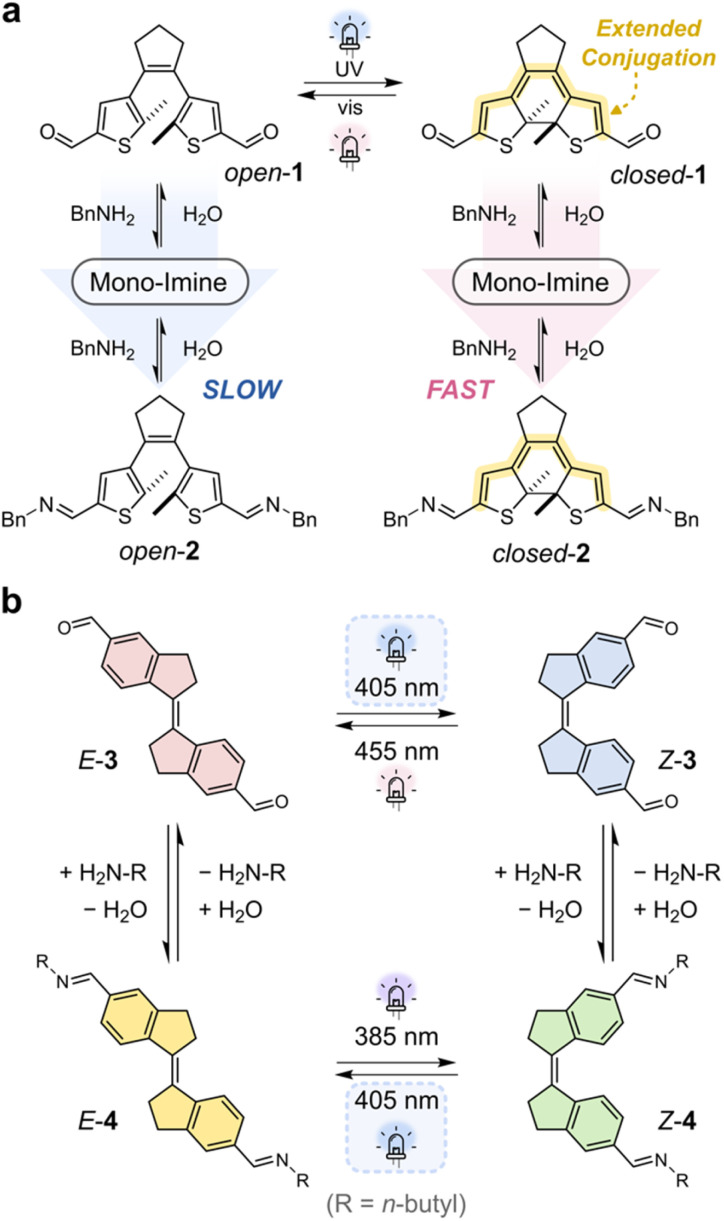
(a) Light-controlled modulation of imine condensation using a diarylethene (DAE) photoswitch, as reported by Hecht and co-workers.^55^ The ring-closed isomer increases electronic conjugation between the aldehyde units, accelerating bis-imine formation relative to the open form. (b) A stiff-stilbene system developed by Wezenberg and co-workers,^84^ in which imine condensation alters the photochemical behavior of the switch. The *E*- and *Z*-isomers favor hydrolysis and condensation, respectively, enabling a reversible cycle under continuous 405 nm irradiation. Such a system could be interesting in the context of achieving a NESS under constant irradiation.

Modulating the electronic properties of an imine *via* a neighboring photoswitch has proven effective in controlling its dynamic covalent behavior. Elegant work by Hecht and co-workers has employed diarylethene (DAE) photoswitches, which undergo a reversible light-induced ring closure to afford a closed state with an increased π-conjugation length (Fig. 5a).^55^ This closed form can be reopened by irradiation with a longer wavelength of light.

In the example shown in Fig. 5a,^55^ the electronic properties of the two aldehyde groups at each end of the DAE core are significantly altered between the open and closed switch states. In the open form, *open*-1, the aldehydes are electronically isolated from one another, thus behave largely independently. However, in the closed form, *closed*-1, conjugation across the DAE core increases electronic communication between the aldehydes, enhancing their electron-withdrawing character. This synergistic activation of the aldehyde units accelerated imine formation by an order of magnitude, enabling light-triggered control over bis-imine assembly. This concept was further applied to develop a self-healing polymer that responds to light input.^55,83^ Crucially, the two photoswitch states are thermally stable (*i.e.*, kinetically trapped), enabling photogated reactivity: the imine condensation rate is fixed into a given state until a second light stimulus is applied to switch the DAE to a different state. In this sense, the entire system—including the thermal imine condensation reaction—functions as a bistable molecular photoswitch, where a distinct equilibrium is achieved for each of the switched states.^9^

The feedback between imine formation and photoswitching can also be bidirectional. Wezenberg and co-workers demonstrated a system where imine formation influenced the photochemical behavior of the switch itself (Fig. 5b).^84^ In their design, a formylated stiff-stilbene switch, *E*/*Z*-3, could modulate the electronics of imine condensation and hydrolysis. The *E*-isomer favored the hydrolysis product, while the *Z*-isomer promoted the imine condensation product, 4. Irradiation of bis-aldehyde *E*-3 at 405 nm produced *Z*-3, which favored imine condensation to yield *Z*-4. Importantly, now *Z*-4 can absorb light at wavelengths suitable for photoisomerization back to the *E*-isomer, affording *E*-4, which in turn shifted the equilibrium back toward hydrolysis and *E*-3. Although a full *in situ* cycling experiment was not reported, this network architecture could, in principle, be driven to a NESS under continuous light irradiation (405 nm), due to the cyclic interconversion between the photochemical and thermal steps (*E*-3 to *Z*-3 to *Z*-4 to *E*-4). However, if the light is not continuously supplied, the system would be driven to a new equilibrium state due to the bistability of the *E* and *Z* isomers (*i.e.*, kinetically trapped). In other words, photoirradiation bridges *E*-3 and *Z*-3 as well as *E*-4 and *Z*-4; without light *E*-3 and *E*-4 are in thermal exchange as is *Z*-3 and *Z*-4. Under the operating conditions of the system, interconversion between the *E*/*Z* isomers is only possible with photoirradiation. Therefore, the irradiation conditions play a key role in the equilibrium behavior exhibited by the system.

A similar design was reported by Feringa and coworkers, who showed how a salicylidene-containing stiff-stilbene molecular motor^85^ could be used to tune the motors operation. Rather than exploiting imine condensation and hydrolysis, the authors showed how keto–enol tautomerization could be used to red shift the motor's UV/vis absorption bands. Specifically, this salicylidene-moiety imparted both temperature and solvent sensitivity to the system, thus influencing the achievable PSSs.

### Light-induced geometric changes to modulate steric strain

**Fig. 6:**
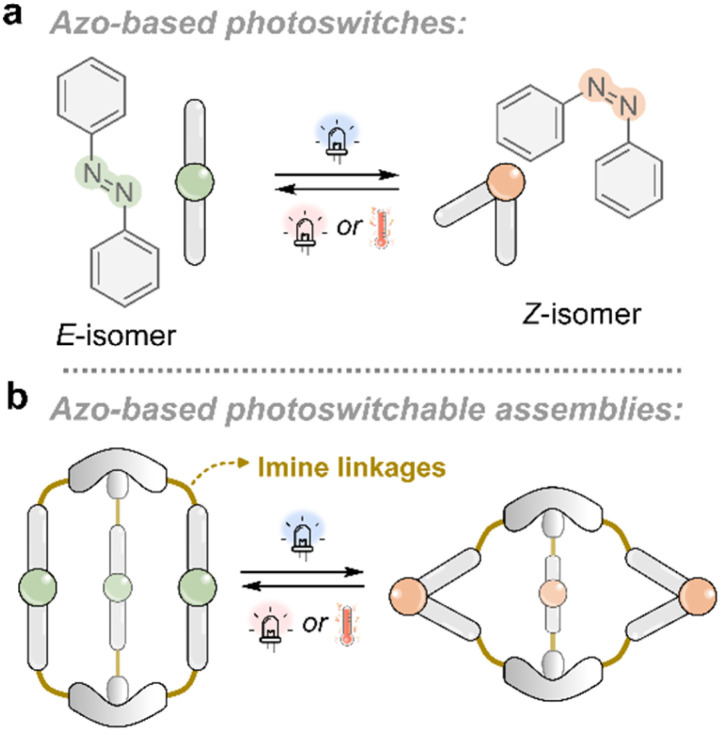
(a) General schematic of an azo-based photoswitch undergoing reversible *E*/*Z* isomerization. (b) Light-induced switching of azobenzene-containing units embedded in a dynamic covalent cage can induce structural changes that modulate the internal cavity size and shape, enabling potential control over host–guest interactions.

A change in molecular geometry upon photoisomerization offers another strategy to perturb imine equilibria. Photoswitches that undergo *E*/*Z* isomerism, such as azobenzenes and their azoheteroarene derivatives (Fig. 6a), are particularly well suited for this purpose. In most cases (apart from the diazocines,^86^ which exhibit the reverse behavior), the relatively linear *E*-isomer is more thermodynamically stable than the more twisted *Z*-isomer.^87,88^ Azo-based photoswitches are especially attractive due to the extensive design rules reported to rationally tailor their photoswitching properties. These include control over the completeness of photoswitching (PSS distribution), wavelength sensitivity, and thermal half-life of the metastable state.^86,88–92^

An elegant example of using light-induced *E*/*Z* geometry changes to bias imine behavior was reported by Schmidt and co-workers in 2022 (Fig. 6).^20^ They combined a rigid, linear dialdehyde unit containing a tetra-fluorinated azobenzene core^93^ with different amines to assemble dynamic imine-based macrocycles and linear oligomeric chains.^20,94^ The fluorinated azobenzene enabled efficient *E*/*Z* photoisomerization with visible light and conferred increased thermal stability to the *Z*-isomer.^89,93,95^ When a flexible ethylenediamine was combined with the dialdehyde in its more linear *E*-isomer, the system favored the formation of linear oligomers. Upon photoswitching the dialdehyde component to the bent *Z*-isomer, the same components yielded discrete macrocycles instead. Interestingly, switching the *Z*-isomers within the macrocycle back to the *E*-isomer triggered imine exchange, re-forming linear oligomers. This behavior reflects a geometric mismatch: the *E*-isomer is incompatible with the macrocyclic structure, possibly due to strain, leading to structural reorganization. Thus, the *E*-form macrocycle represents an OOE state, which returns to equilibrium *via* imine exchange to the thermodynamically favored oligomeric structures. In this case, the high thermal stability of the *Z*-isomer towards relaxation to the *E*-state renders the system kinetically trapped, resulting in the imines re-equilibrating to a new local minimum.

Interestingly, when the authors employed a more rigid diamine,^20^*trans*-1,2-diaminocyclohexane—which imposes greater geometric constraints on the assembly—only macrocycles were observed. With the *E*-isomer of the dialdehyde, the system selectively formed a single dominant size of macrocycle, a 3mer. In contrast, using the *Z*-isomer afforded a distribution of macrocycles of varying sizes. When the *E*-isomer-derived 3mer macrocycle was irradiated to generate the *Z*-isomer *in situ*, the system responded by redistributing into a mixture of macrocycles of varying size. This indicates that the 3mer macrocycle formed from the *E*-isomer-dialdehyde is OOE when converted to the *Z*-isomer and relaxes by imine exchange to structures with reduced steric strain. Thus, the *E*/*Z* isomerization of the azobenzene photoswitch introduces strain mismatches within the macrocyclic framework, driving the system away from its thermodynamic minimum and triggering redistribution. While the imine is electronically conjugated to the photoswitch, the dominant effect here appears to arise predominantly from steric and geometric changes, rather than electronics.

Steric strain can also be induced by the winding motion achieved by a molecular motor. For example, Giuseppone and co-workers demonstrated a molecular whirligig where the motor unit could wind polymer strands into a more tensed, state.^96^ However, the impact of such light-induced strain buildup on imine equilibrium reactions can be difficult to predict, as shown by the work of Kathan, Schalley, Feringa and co-workers.^11^ In their system, a light-powered molecular motor was used to twist two flexible strands—each attached to the rotor and stator—into mechanically strained macrocycles that incorporate an imine bond. Upon photoirradiation, the motor incrementally winds the strands, increasing the number of crossings of these chains within the structure. This light-fueled winding drives the system into progressively higher energy states. To investigate the effect of strain and steric hindrance on imine exchange, a small competing amine was introduced to open the macrocycle, *via* transimination. The system responded to this perturbation by converting into states with fewer crossings. The rate of imine exchange was unaffected by the degree of winding: the kinetics were comparable for both the lowest and highest crossing-number structures. However, as noted by the authors,^11^ the reverse process—re-forming the highly wound macrocycle from the opened strands—was not observed, indicating that this state represents a high energy-state that is thermodynamically disfavored and only accessible through the light-fueled winding. Overall, the wound system slowly unraveled over time, constituting a NESS when operated under constant irradiation.

### Perturbing self-assembled capsules OOE

Interest in integrating imine-based capsules with photoswitchable units date back to the 1990s.^26^ In 1991, the groups of Bauer and Vögtle reported imine cages formed by condensing tris(2-aminoethyl)amine (TREN) with azobenzene-containing dialdehydes.^26^ Nearly three decades later, interest in these structures resurfaced, and they have since been reinvestigated and further developed by several groups.^20,21,25,97–100^

As schematically illustrated in Fig. 6a, light-induced *E*/*Z* isomerization of the azobenzene units imparts structural changes to the overall architecture (Fig. 6b). This approach enables modulation of the internal cavity size and geometry in response to light. In particular, imine-based metal-templated subcomponent self-assembled cages by Nitschke and co-workers have demonstrated light-induced modulation of guest binding for a variety of different guests including anions,^101^ steroids^102^ and metal-ions.^103^ Metal-templated imine-based helicates have also recently shown ratcheting behavior.^104^ In most of these light-responsive cages, the imine bonds remain under thermodynamic control. That is, the structural response is primarily a change in the shape of the capsule, rather than a light-driven shift in the imine formation or exchange equilibrium.

**Fig. 7:**
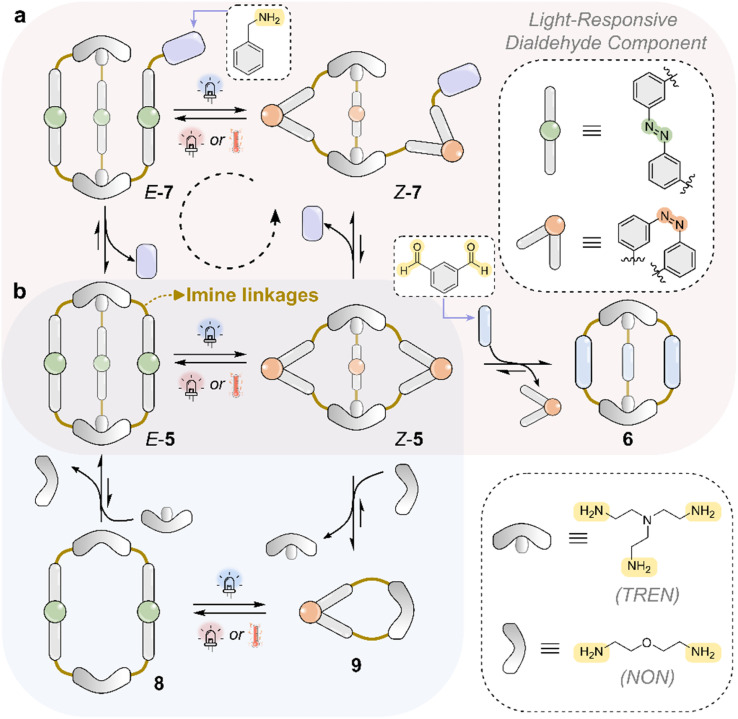
Coupling of azobenzene *E*/*Z* isomerization with imine transimination in a self-assembled cage system developed by Feringa and co-workers,^95,96^ based on an azo-containing dialdehyde and either a trisamine (TREN) or a diamine (NON). (a) Light-induced *E* → *Z* isomerization of cage 5 introduces geometric strain,^95^ triggering transimination of the imine cage with a competing amine (violet) and resulting in cage opening. Subsequent *Z* → *E* isomerization of 7 restores the geometry needed for cage reformation, reforming cage 5. As the cycle requires switching between energy surfaces using different wavelengths of light, this system operates *via* an energy ratchet mechanism. (b) When using NON instead of TREN, reversible light-controlled transitions are observed between cage and macrocyclic architectures, governed by the isomerization state of the azobenzene units.^96^

An example of a photoswitchable imine-based capsule in which light directly perturbs the imine bond OOE was reported in 2022 by Feringa and co-workers.^97^ They developed an azobenzene-based imine cage system, *E*-5, that couples light-induced *E*/*Z* isomerization with dynamic covalent chemistry to achieve reversible light-driven transformations and access OOE states (Fig. 7a).^97^ Upon UV light irradiation of *E*-5, *E*-to-*Z* isomerization of the azobenzene units generates *Z*-5, which introduces geometric strain that destabilizes the cage and promotes opening *via* transimination with added benzylamine to afford *Z*-7. This process is reversible: thermal or visible-light-induced *Z*-to-*E* isomerization generates *E*-7, which can then undergo transimination to reform the original cage *E*-5, completing a light-fueled opening/closing cycle.

Importantly, this coupling of photochemical and dynamic covalent transformations allows the population of higher-energy species, such as *Z*-5 and *E*-7, to equilibrate to new local minima after photoisomerization (kinetically trapped photoisomers). These species are otherwise inaccessible under purely thermodynamic conditions in a stepwise manner. Alternatively, rather than using benzylamine, *Z*-5 could transform into 6, liberating the *Z*-dialdehyde building block of the cage to reduce unfavorable steric strain.

In 2024, this concept was further extended with a system capable of reversible, light-driven interconversion between multiple self-assembled architectures—including cages and macrocycles—*via* photoinduced geometric strain (Fig. 7b).^98^ Using the same azobenzene-containing dialdehyde that forms either macrocycles or cages depending on the amine component, the authors showed that *E*/*Z* photoisomerization can control dynamic reassembly between [3 + 2] cages and [*n* + *n*] macrocycles. As before, *E*-to-*Z* isomerization of *E*-5 introduces strain in *Z*-5, destabilizing the cage and promoting constitutional exchange with a competing amine (NON) to form macrocycle *Z*-9. This *Z*-9 macrocycle could then be photoisomerized back to the *E*-form, triggering a structural change into the [2 + 2] macrocycle *E*-8. Subsequent transimination allows regeneration of the original cage *E*-5, thus completing a fully reversible, light-driven structural cycle. Notably, the study demonstrates seven such cage-to-macrocycle transitions, generating otherwise inaccessible higher-energy architectures.

In these examples of Feringa and co-workers, the ability of light-responsive cage systems to operate directionally out of equilibrium, rather than randomly with no net direction, can be understood through the framework of a ratcheting mechanism.^9,10^ In such mechanisms, a driving process—such as light-induced isomerization into a metastable state—supplies energy, while a driven process harnesses this energy to directionally move the system away from its equilibrium distribution. This coupling between the driving and driven processes displaces the system from its equilibrium distribution, either by shifting it against a thermodynamic gradient or by stabilizing populations that would be disfavored under equilibrium conditions.

Two types of light-fueled ratcheting mechanisms are typically considered: energy ratchets and information ratchets.^105^ In an energy ratchet, the system is repeatedly cycled between different potential energy surfaces by externally switching conditions—such as the wavelength of light—or by spatially separating these illumination conditions. This repeated cycling, independent of the state of the system,^9,10^ enables net flux through sequential energetically downhill steps, often described as “power strokes”. In contrast, an information ratchet generates directionality *via* state-dependent interactions with the energy source, such that the system's response to light is governed by its current configuration. This asymmetry in photochemical behavior and kinetics breaks detailed balance and allows the system to achieve net flux under continuous irradiation.

In the cage system reported by Feringa,^97,98^ the full operation of the light-fueled cycle requires sequential photoisomerization steps: first, *E*-5 to *Z*-5 to induce transimination and cage opening, followed by *Z*-7 to *E*-7 to promote cage reformation. Because the system relies on externally applied changes in irradiation conditions to complete the cycle, it is best described as operating *via* an energy ratchet mechanism.^9,10^ By contrast, the previously discussed light-driven system developed by Wezenberg appears to be more appropriately described as an information ratchet when operated under constant irradiation with 405 nm light.^84^ In that case, the markedly different photochemical properties and imine exchange behavior of the *E*- and *Z*-isomers allow for a complete, directional cycle to emerge under continuous 405 nm irradiation (*E*-3 to *Z*-3 to *Z*-4 to *E*-4).

### Come together: integrated photoswitchable imines

Whereas the modular strategy couples a photoswitchable unit to an imine-containing dynamic covalent system, the integrated approach embeds photoresponsiveness directly within the imine bond itself. This strategy relies on inherently photoswitchable imines, combining photoswitching and dynamic-covalent behavior within a single bond.^35,106,107^ Although only recently developed to the point where such systems can be reliably studied,^106,108,109^ the integrated approach presents promising opportunities for light-driven dynamic covalent chemistry.

Historically, aldimine (R^1^HC=NR^2^) photoswitches were overlooked as competitive photoswitches due to their reportedly poor photoswitching performance compared to azo-based counterparts.^110,111^ Challenges included incomplete photoconversion to the metastable *Z*-isomer (typically <50% being the *Z*-isomer), short thermal half-lives of the *Z*-isomer (*t*_1/2_ < 60 s at 20 °C), and the need for UV light (≤365 nm) to induce photoisomerism.^106^ This combination of limitations presented a significant challenge in both characterizing and exploring the properties of the metastable *Z*-isomer.

**Fig. 8:**
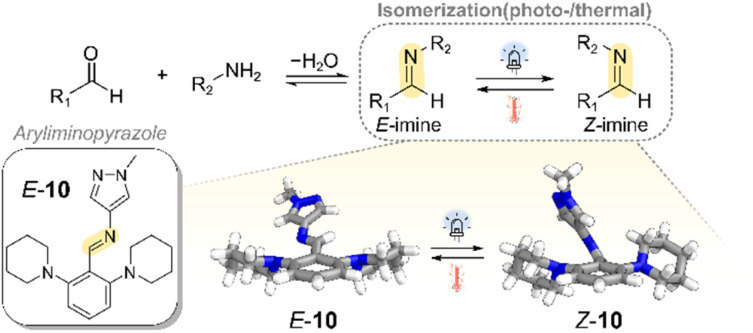
Example of a photoswitchable aryliminopyrazole (AIP) imine.^103^ The crystal structures of both the *E*- and *Z*-isomers highlight the geometric changes associated with photoisomerization,^109^ confirming reversible light-induced *E*/*Z* switching at the imine bond. These systems can exhibit quantitative *E*-to-*Z* conversion under visible light and feature *Z*-isomer thermal half-lives exceeding one day at room temperature.^109^

Our group recently developed a new class of imine-based photoswitches—the aryliminopyrazoles (AIPs)—that address these former limitations (Fig. 8).^106,112^ AIPs undergo efficient *E*/*Z* photoisomerization under visible light (up to 470 nm), reaching photostationary states with >95% of the imine as the *Z*-isomer. Unlike the earlier investigated arylimines, AIPs display a significantly improved thermal stability of their *Z*-isomer, with *t*_1/2_ values exceeding one day at room temperature. This property enabled structural confirmation of *E*/*Z* photoisomerism *via* single-crystal X-ray diffraction (Fig. 8), marking the first crystal structure of a light-induced metastable *Z*-imine in the CCDC.^112^ Together, these advances in photoswitching efficiency, thermal stability of the metastable *Z*-isomer, and structural characterization have enabled reliable investigation of light-responsive dynamic covalent behavior in these integrated systems.

**Fig. 9:**
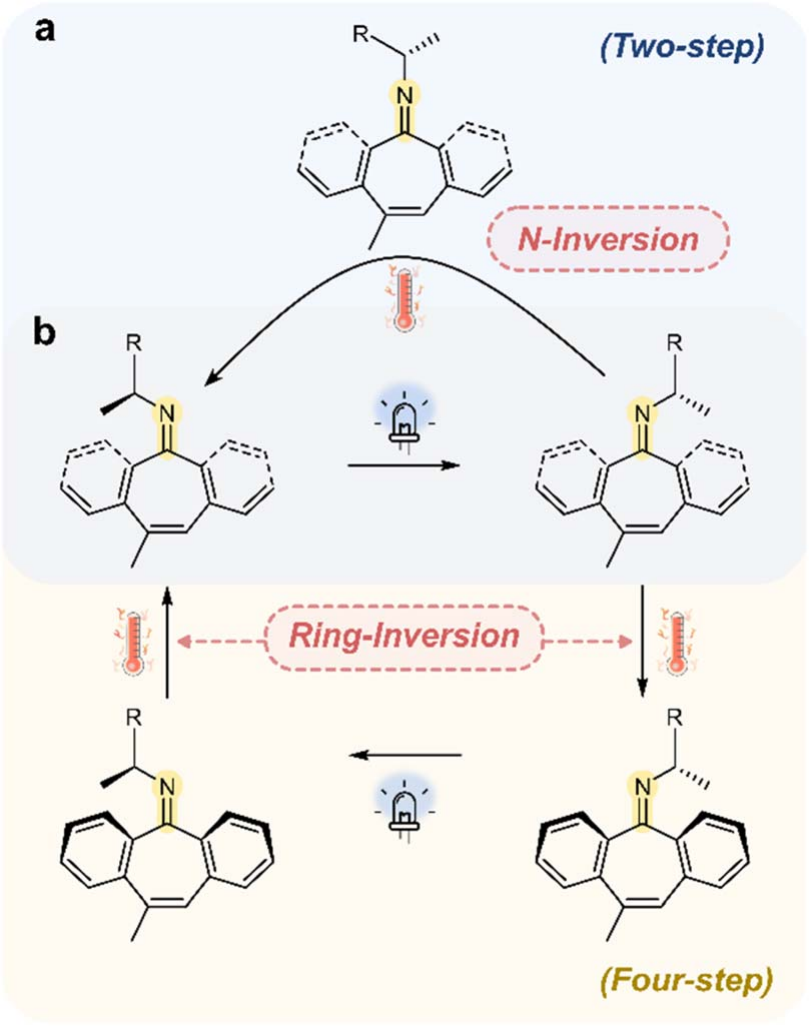
(a) A two-step molecular motor that operates around a C=N bond. The operation of the motor follows a photoinduced directional rotation, followed by a thermal *N*-inversion step. (b) A four-step motor, consisting of two photoinduced rotation steps and two thermal ring-inversion steps.

It should be noted that Lehn and coworkers introduced ketimines (R^1^R^2^C=NR^3^) as light-driven molecular motors that couple a photochemical out-of-plane rotation about the C=N bond with a thermal inversion (Fig. 9).^18,107^ A preferred directionality of rotation is achieved by incorporating a stereogenic center. When the thermal step proceeds *via* linear *N*-inversion, the motor operates through a two-step cycle, requiring only one photon to complete a full cycle. If instead the thermal relaxation step follows a ring-inversion pathway, the motor exhibits a four-step cycle, consisting of two photoinduced rotations and two thermal inversion steps. This concept has since been further explored by other groups.^113–115^ Notably, in all these examples, while the imines are inherently dynamic covalent bonds, their photoresponsive behavior is not coupled to an exchange process. Mechanistically, and in contrast to the stiff-stilbene motors mentioned previously, these imine motors are able to undergo a linear inversion pathway due to the presence of the nitrogen atom in the photochromic bond. During the inversion, the nitrogen must rehybridize from sp^2^ to sp to achieve the required linear geometry; this is not possible for the sp^2^ carbon atoms of the photochromic C=C bond in stilbene.

### Driving transimination reactions out of equilibrium using light

We recently demonstrated that photoswitchable imines can couple photoisomerization with transimination to drive a dynamic covalent system into an OOE distribution of imines that lies energetically uphill.^12^

Using AIP photoswitches with aniline as a competing amine, the system underwent transimination to afford a mixture of imines 11 and 12.^12^ While imine 11 is more thermodynamically stable, light irradiation shifted the imine composition toward imine 12, indicating an endergonic transformation fueled by light. Kinetic studies revealed that the metastable *Z*-isomer is more reactive toward transimination than the *E*-isomer. In addition, the two imines display distinct photoswitching properties: imine 11 reaches a photostationary state (PSS) with ∼95% *Z*-isomer, whereas imine 12 reaches only ∼30%.

**Fig. 10:**
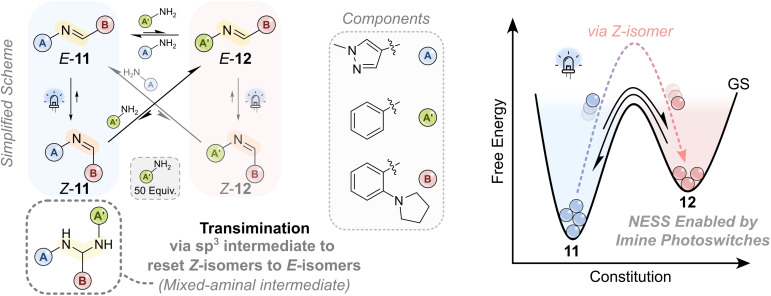
Simplified reaction network between photoswitchable imines and a schematic free energy diagram of the systems constitution.^12^ The system contains two amines (A and A′) and an aldehyde (B); aniline (A′) is used in large excess (50 equiv.) as its corresponding imine is thermodynamically disfavored. In the dark, an equilibrium is established between imines 11 and 12*via* transimination through a sp^3^-hybridised aminal intermediate. Upon 405 nm irradiation, both imines undergo *E*/*Z* photoisomerization: imine 11 reaches a PSS with ∼95% *Z*-isomer, while imine 12 reaches only ∼30% *Z*-isomer. The *Z*-isomers also participate in transimination but with distinct composite rate constants compared to their *E*-isomer counterparts. Under continuous irradiation, these differences in both switching properties and kinetics result in a NESS. As the system's reactivity and photoswitching behavior depends on its current state, the resulting net flux under constant light is consistent with an information ratchet mechanism. Notably, the reverse *Z*-to-*E* transimination *via* the aminal intermediate is disfavored, enforcing directionality. The aminal intermediate itself serves as a crucial branching point, funneling the system toward distinct imine products.^12^

This combination of isomer-dependent reactivity and distinct PSS distributions leads to a net accumulation of the less stable imine under continuous irradiation, establishing a light-driven NESS. Although two competing cycles are accessible in the system (*E*-11 to *Z*-11 to *E*-12 to *E*-11 and the other *E*-12 to *Z*-12 to *E*-11 to *E*-12), the difference in the photoswitching properties between imines 11 and 12 results in a net flux of the system as shown in Fig. 10. Specifically, the *Z*-isomers of 11 and 12 display different reactivity and are produced in unequal amounts under photoirradiation, generating a kinetic bias that favors directional cycling. These features are consistent with an information ratchet mechanism: directionality arises from the intrinsic reactivity and photoresponse of system states, allowing autonomous operation under constant light irradiation.

An important and interesting feature of this system is that transimination not only forms a new imine, but also simultaneously resets a *Z*-imine to an *E*-imine *via* the sp^3^ hybridized mixed aminal intermediate.^12^ Although the reverse transformation, *i.e.*, *E*-imine to mixed aminal to *Z*-imine, is thermally possible, its contribution is negligible under experimental conditions. This unidirectional resetting of the isomer state effectively “closes the door behind itself,” ensuring that once a *Z*-imine undergoes transimination to the *E*-isomer, it can only be converted to a *Z*-isomer by photoexcitation. This contrasts with the behavior in modular systems described in approach A, where transimination is not inherently coupled to *Z*-to-*E* relaxation, though in specific cases—such as in Wezenberg's system when operated under continuous irradiation—^84^ this coupling can still arise due to the distinct switching behavior of the imines involved. Moreover, even if the *E*/*Z* isomers of imine 11 could be designed to be kinetically trapped, the process of transimination results in resetting the *Z*-isomer back to the *E*-isomer without requiring absorption of a photon. Thus, embedding the photoswitching function directly into the dynamic imine bond appears especially well suited for designing autonomous, light-driven information ratchets.

### Driving non-photoresponsive transimination reactions out of equilibrium using light

Using light to perturb a reaction that is not itself photoresponsive, at the wavelength used, requires the coupling of a photochemical process to a secondary perturbation that the non-photoresponsive reaction can respond to. Such coupling can give rise to light-fueled reaction cascades, inspired by those found in biological systems.^13^

**Fig. 11:**
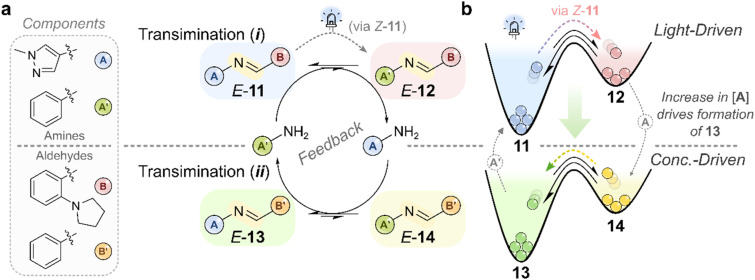
(a) A light-fueled imine network in which a photoswitchable transimination reaction (i) perturbs a coupled, non-photoresponsive transimination reaction (ii).^13^ The system comprises two amines (A and A′) and two aldehydes (B and B′) in a 1 : 3 : 1 : 1 stoichiometry. Imines derived from the *ortho*-pyrrolidine aldehyde (B) are photoswitchable under 405 nm light, whereas those formed from benzaldehyde (B′) are not. Upon irradiation, photoisomerization shifts transimination (i) OOE, increasing the population of imine 12 and releasing more of the more nucleophilic amine A. This excess A then reacts with the non-photoresponsive imine 14 in (ii) to form imine 13 and release amine A′, which is in turn consumed by the light-driven cycle. The resulting feedback loop couples the two transimination reactions and drives the entire system—including the non-photoresponsive reaction—into a NESS under continuous irradiation. (b) Schematic energy landscape of the system, illustrating how transimination (i) is driven out of equilibrium by light, while transimination (ii) is perturbed from equilibrium by the resulting change in amine population.^13^

Building on our previous demonstration that photoswitchable imines can drive dynamic covalent systems OOE, we recently established a light-fueled reaction cascade capable of driving a non-photoresponsive transimination OOE (Fig. 11).^13^ In this system, a dynamic covalent network of four interconverting imines, 11–14,—formed from two amines and two aldehydes—was prepared, resulting in two distinct transimination reactions, (i) and (ii), Fig. 11a. In isolation, transimination (i) responded to 405 nm irradiation, while the other did not. However, when both equilibria were combined in a single system, photoisomerization of the responsive imines perturbed not only their own equilibrium but also the coupled, non-photoresponsive transimination, driving the entire system into a NESS.^13^

The mechanism relies on the light-driven transimination of imine 11 to 12 using a small excess of aniline (A′, 2 equiv.), which simultaneously liberates amine A. Amine A is more nucleophilic than aniline and reacts with the non-photoresponsive transimination, converting imine 14 into 13 and regenerating aniline. The production and consumption of the two amine components across both transimination reactions establishes a feedback loop that amplifies the system's departure from thermodynamic equilibrium. Under these conditions, the system is perturbed significantly farther from equilibrium than expected based solely on the presence of 2 equiv. of aniline.^12,13^ This enhanced displacement is attributed to the coupled feedback between the two transimination reactions, which effectively reinforces the perturbation—analogous to two pedals of a bicycle, alternately pushing the system uphill. Interestingly, the preference to forming imine 12 and 13 under photoirradiation can also be explained in terms of agonistic and antagonistic relationships between the components in the [2 × 2] network (Fig. 2). What is different in this example to that typically shown in the literature,^71^ is that this new network state is not a new thermodynamic minimum, but a NESS.

This example extends the scope of light-responsive dynamic covalent chemistry by demonstrating that photoswitchable bonds can drive entire coupled reaction cascades, not just individual equilibria, in homogeneous solution. Such a strategy could be applied to more complex systems, such as supramolecular assemblies or host–guest networks. More broadly, designing systems with multiple, interlinked light-controlled dynamic covalent reactions—whether cooperative or competitive—offers a route to constructing synthetic networks capable of hierarchical energy transduction, mimicking key features of biological reaction cascades.^116–118^

## Conclusions and outlook

The examples discussed illustrate how light-induced geometric or electronic changes can be harnessed to drive dynamic-covalent imine systems away from equilibrium. Two general design strategies have emerged: modular and integrated. The modular approach maintains more of a clear separation between the photoswitch and the dynamic covalent bond, allowing each to be optimized more independently using established design principles. When the photoswitch and imine are only weakly coupled, these systems appear better suited to operate as energy ratchets: photoisomerization can perturb a coupled equilibrium, but completing the cycle typically requires a change in irradiation conditions to reset the switch back to its starting state. In contrast, the integrated approach embeds photoresponsiveness directly within the imine bond, creating a tighter coupling between switching and reactivity. While this makes independent tuning more difficult, it introduces a state-dependent response that can produce directional behavior under continuous irradiation, consistent with operation as an information ratchet. Moreover, in transimination reactions, passage through a tetrahedral mixed-aminal intermediate provides a particularly effective mechanism for achieving unidirectionality—specifically by resetting the photoswitch to the thermodynamically more favored *E*-isomer.

A recent perspective article from Credi and co-workers have outlined key challenges in this area,^1^ some of which have recently begun to be addressed.^13^ In the context of the imine-based systems discussed here, further questions from our own perspective include:

(1) *Host–guest chemistry*: Can light-driven imine exchange add value to host–guest systems? Photoresponsive cages have been used to modulate guest binding; could dynamic imine exchange add value in terms of guest selectivity or kinetics?

(2) *New (emergent) behaviors*: What new behaviors and functions—particularly those performing useful work—can emerge from these NESSs?

(3) *Driving force*: How far can light-driven transimination be pushed from equilibrium? Both theoretical and experimental tools are needed to better understand the limits of this perturbation and the design parameters that control it.

(4) *Autonomy*: What strategies are most effective for autonomous operation? Information ratchets operate autonomously under constant irradiation, but energy ratchets can also be made autonomous through experimental design, for example by using broad-spectrum light or spatially separated stimuli (as demonstrated in U-tube setups).^119,120^ A comparison of the relative merits would be useful here.

To conclude, imine-based systems under thermodynamic control remain a highly active and fruitful area of research. The development of light-driven systems that operate out of equilibrium is still in its early stages, but we anticipate a bright and more expansive future. After all, people don't tend to climb mountains to admire the base—the most compelling states often lie far from equilibrium.

## Author contributions

The focus of the perspective was conceived by both J. W. and J. L. G. J. W. prepared an initial draft that was edited by both J. W. and J. L. G. J. L. G. acquired funding and supervised the work.

## Conflicts of interest

There are no conflicts to declare.

## Note added after first publication

This article replaces the version published on 8th September 2025, where the title contained a typographical error, this has now been resolved.

## Data Availability

No primary research results, software or code have been included, and no new data were generated or analyzed as part of this perspective article.
